# Supplementary material for: Phthalate Exposure Pattern in Breast Milk within a Six-Month Postpartum Time in Southern Taiwan
Source: Int J Environ Res Public Health. 2021 May 26;18(11):5726. doi: 10.3390/ijerph18115726 (PMC8198263; doi:10.3390/ijerph18115726)
Supplement: Supplementary file 1 [file ijerph-18-05726-s001.zip › ijerph-1184058-supplementary.pdf]

Phthalate Exposure Pattern in Breast Milk Within a Six-Month Postpartum Time in Southern Taiwan

Shen-Che Hung†, Ting-I Lin†, Jau-Ling Suen, Hsien-Kuan Liu, Pei-Ling Wu, Chien-Yi Wu, Yu-Chen S.H. Yang, San-Nan Yang and Yung-Ning Yang*

Supplementary Table S1. LOQ, LOQ, and linear range of phthalate metabolites

	LOD[1] (ng/mL)	LOQ[2] (ng/mL)	linear range (ng/mL)	correlation coefficient
**MEHHP**	0.15	0.5	0.5~1000	$R^2 = 0.9998$
**MECPP**	0.15	0.5	0.5~1000	$R^2 = 0.9999$
**MEOHP**	0.15	0.5	0.5~1000	$R^2 = 0.9997$
**MEHP**	0.15	0.5	0.5~1000	$R^2 = 0.9988$
**MBZP**	0.3	1	1~1000	$R^2 = 0.9999$
**MIBP**	0.6	2	2~1000	$R^2 = 0.9985$
**MBP**	0.6	2	2~1000	$R^2 = 0.9994$
**MCMHP**	0.3	1	1~1000	$R^2 = 0.9994$

[1]Limit of detection (LOD) was calculated at signal-to-noise ratio (S/N) = 3.

[2]Limit of quantitation (LOQ) was calculated at signal-to-noise ratio (S/N) = 10.
